# Muscle specific miRNAs are induced by testosterone and independently upregulated by age

**DOI:** 10.3389/fphys.2013.00394

**Published:** 2014-01-23

**Authors:** Søren Nielsen, Thine Hvid, Meghan Kelly, Birgitte Lindegaard, Christine Dethlefsen, Kamilla Winding, Neha Mathur, Camilla Scheele, Bente K. Pedersen, Matthew J. Laye

**Affiliations:** ^1^Department of Infectious Diseases, The Centre of Inflammation and Metabolism and the Centre for Physical Activity Research, Rigshospitalet, University of CopenhagenCopenhagen, Denmark; ^2^The Buck Institute for Research on AgingNovato, CA, USA

**Keywords:** miRNA, skeletal muscle, myomiRs, aging, gender, exercise

## Abstract

Age dependent decline in skeletal muscle function leads to impaired metabolic flexibility in elderly individuals. Physical activity and testosterone treatment have proven efficient strategies for delaying this condition. However, a common molecular pathway has not been identified. Muscle specific miRNAs (myomiRs) regulate metabolic pathways in skeletal muscle, are regulated by physical activity, and have response elements for testosterone in their promoter region. We therefore hypothesized that myomiRs would be regulated in skeletal muscle during aging. We further investigated any potential gender-dependent regulation of these miRNAs. We found that the myomiRs miR-1, miR-133a, and miR-133b were increased in skeletal muscle of elderly men compared to younger men. In addition, miR-133a/133b expression was markedly higher in women compared to men. Elimination of circulating testosterone in men was associated with lower levels of miR-133a and miR-133b. A positive regulatory effect of testosterone on miR-133a/133b expression was confirmed in castrated male C57BL/6J mice and in a model of primary human myocytes. Yet, an improvement of fitness level in the testosterone depleted men resulted in a down-regulation of miR133a/b. In conclusion, alterations in fitness level and circulating testosterone seem to represent two independent regulatory events where testosterone is a specific regulator of miR-133a/b expression.

## Introduction

The normal aging process involves a decline in skeletal muscle function. Several metabolic alterations are associated with aging, including impaired glucose, lipid and protein metabolism, subsequently leading to metabolic inflexibility and loss of skeletal muscle function (Ramamani et al., 1999; Gibney et al., 2005; Frederiksen et al., 2012).

Age related metabolic dysregulation is a multifactorial and gender specific process, involving decreased physical activity and alterations in steroid hormonal levels (Booth and Zwetsloot, 2010; Kelly and Jones, 2013). Importantly, physical activity delays and/or diminishes the age related metabolic alterations independently of steroid hormones (Yarasheski, 2003). The steroid hormone testosterone is synthesized mainly in the testis in men and to a minor degree in the ovaries in women and plays a key role in carbohydrate, fat, and protein metabolism in tissues expressing the androgen receptor (AR), including skeletal muscle (Ramamani et al., 1999; Frederiksen et al., 2012; Phillips et al., 2012). In men, circulating testosterone levels peak around age 30–40 and gradually decrease thereafter. At the age of 75, testosterone levels are approximately decreased by 30%, compared to peak levels (Deslypere and Vermeulen, 1984; Muller et al., 2003). In adult women, circulating testosterone concentrations are approximately 10 times as low as in men (Torjesen and Sandnes, 2004) and likewise decrease with age (Yasui et al., 2012). Circulating testosterone can be bound to carrier proteins but also exist in an unbound form, which is normally referred to as free testosterone. The molecular mechanisms by which circulating testosterone controls muscle metabolism are not fully clear.

The canonical pathway through which free testosterone acts is by diffusing through the cell wall and binding to cytosolic AR's, which translocate to the nucleus and bind to DNA as dimers. Nuclear AR binds to androgen response elements (AREs) within genomic promoter regions, where it can regulate gene transcription (Heemers and Tindall, 2007). Interestingly, AREs have been identified in the promoter regions of the non-coding regulatory group of RNAs called microRNAs (miRNAs) (Delic et al., 2010). Mature miRNAs are typically 20–22bp in length, and function through physical association with the 3′UTR of a mRNA, either degrading the mRNA or causing ribosomal stalling to reduce protein translation (Bartel, 2004). A single miRNA can regulate the expression of hundreds of mRNAs and proteins (Lim et al., 2005; Baek et al., 2008) and these small RNAs therefore play a crucial role in many aspects of biology, including cell cycle regulation, developmental processes and metabolic regulation (Chen et al., 2006; Zhao et al., 2007; Gallagher et al., 2010; Lujambio and Lowe, 2012). miR-1, miR-133a, miR-133b, and miR-206 belong to a group of muscle specific miRNAs (myomiRs) crucial for the regulation of skeletal muscle development and function (Chen et al., 2006; van Rooij et al., 2008). Five active ARE motifs have been experimentally identified and verified in the promoter region near the co-transcribed miR-206/miR-133b locus (Wyce et al., 2010). ARE motifs near the miR-1/miR-133a loci, have not yet been identified. Importantly, in addition to steroid hormones, myomiR loci are regulated by muscle specific transcription factors referred to as Myogenic Regulatory Factors (MRFs) (Chen et al., 2006; Rao et al., 2006; Sweetman et al., 2008). MRFs are vital in the process of coordinating skeletal muscle development and repair (Ramamani et al., 1999). Interestingly, AREs are also found within the promoters for two of the four MRFs, myogenic differentiation 1 (MYOD1) and myogenin (MYOG), suggesting that androgens may indirectly alter myomiR transcription (Dubois et al., 2012). Taken together, transcriptional regulation of myomiRs might be controlled directly and/or indirectly by androgens.

We have previously shown that the expression of all four myomiRs decreases in response to an endurance training program suggesting that these myomiRs are important for metabolic adaptation to endurance exercise (Nielsen et al., 2010). Furthermore, a study by Drummond and co-workers examined myomiR expression patterns in muscle in relation to aging (Drummond et al., 2008b). No differences in basal expression of mature functional myomiRs between elderly and younger men were detected. However, expression of myomiRs in response to resistance exercise and ingestion of essential amino acids was different between elderly and young men. Therefore, anabolic resistance in aged skeletal muscle seems to have a different response mechanism to anabolic stimuli. In addition, myomiR expression is altered in several mice and human models investigating skeletal muscle atrophy and hypertrophy (Huang et al., 2011; Ringholm et al., 2011) and miR-206 is specifically down-regulated in muscle of patients with Amyotrophic lateral sclerosis (Russell et al., 2012).

Interestingly, one validated target of miR-133a/b is the insulin-like growth factor-1 receptor (IGF-1R) in skeletal muscle (Huang et al., 2011), making miR-133a/b a likely regulator of growth factor signaling through the AKT signaling pathway (Schiaffino and Mammucari, 2011). Furthermore, it has been shown that miR-133a and miR-206 are lower in skeletal muscle of people with type 2 diabetes (Gallagher et al., 2010). Therefore, several pieces of evidence indicate that myomiRs are likely to be involved in the process of maintaining metabolic flexibility in skeletal muscle.

Thus, our aim was to investigate whether myomiRs are dysregulated in aged skeletal muscle. Based on previous observations that myomiR expression decreases with increased aerobic fitness and that aerobic fitness decreases with age (Booth and Zwetsloot, 2010; Nielsen et al., 2010; Keller et al., 2011), we hypothesized that skeletal muscle of older individuals would have higher myomiR expression. In addition, given that men and women have markedly different testosterone levels and given a recent report showing that the transcriptome in aged muscle differs markedly between genders (Liu et al., 2013), we further investigated potential differences in myomiR expression between men and women. Finally, we examined whether testosterone, a hormone delaying the age dependent decline in muscle function, regulates myomiR expression.

## Methods

### Ethical approval

The human studies (*in vivo* study 1 and 2) were approved by the local Ethical Committee of Copenhagen and Frederiksberg (KF 01-141/04) (H-4-2009-102) and were performed in accordance with the Declaration of Helsinki. The purpose of the study and its possible risks and discomforts were explained to the participants before their written consent was obtained.

### *In vivo* study 1

Nine older men and nine older women participated in the study. Before inclusion in the study, a medical examination with blood test screening, maximal oxygen consumption test (VO_2_max), and oral glucose tolerance test were performed. Nine younger men and nine younger women with similar body mass indexes (BMIs) as the elderly were recruited as control groups. The control group underwent the same examinations and tests as the two older groups before inclusion. Subjects were excluded from the study if they had any infections 2 weeks before the study or if they were diabetic or had impaired glucose tolerance. Subject characteristics are listed in Table 1. All subjects arrived in the morning in a fasted state to the lab.

**Table 1:** ***In vivo* study 1**.

	**Young women (*n* = 9)**	**Elderly women (*n* = 9)**	**Young men (*n* = 9)**	**Elderly men (*n* = 9)**	**Two-way ANOVA**
Age	23 ± 1.5^****^	68.1 ± 2.2	21.6 ± 1.8^****^	68.1 ± 2.2	^****^(Age)
Weight (kg)	63.9 ± 7.8	64.4 ± 8.5	77.1 ± 11.2	81.2 ± 8.7	^****^(Gender)
Height (m)	1.7 ± 0.1^§§^	1.7 ± 0.1^§§^	1.8 ± 0.1^§§§§^	1.8 ± 0.1	^****^(Gender)
**BODY COMPOSITION**					
BMI	22.3 ± 2.4	23.7 ± 2.7	23.1 ± 2.8	24.6 ± 2.8	
Lean body mass (kg)	40.6 ± 5.5^§§§§^	39.8 ± 2.7^§§§§^	59.8 ± 5.9	58.6 ± 5.0	^****^(Gender)
Fat mass (kg)	19.7 ± 4.1	21.5 ± 7.1	13.7 ± 8.3	18.8 ± 7.1	
**FITNESS**					
VO2 max (l/min/kg)	35.0 ± 4.1^§^	27.7 ± 6.5	45.9 ± 8.1^****^	28.2 ± 6.2	^****^(Age)
Fitness index (l/min/lbm)	0.9 ± 0.05	0.7 ± 0.05^§^	0.8 ± 0.1^***^	0.05 ± 0.03	^****^(Age)
					^**^(Gender)
**HORMONES**					
Testosterone (nmol/l)	1.8 ± 0.4^§§§§^	0.6 ± 0.3^§§§§^	24 ± 7.0^*^	14.4 ± 7.0	^****^(Gender)
					^*^(Age)
Estradiol (nmol/l)	0.5 ± 0.5^***^, ^§§^	0.04 ± 0.003^§§§^	0.1 ± 0.02^*^	0.1 ± 0.03	^*^(Gender)
					^**^(Age)
**GLUCOSE METABOLISM**					
Glucose (mmol/l)	4.7 ± 0.2	5.0 ± 0.2	4.8 ± 0.1^**^	5.4 ± 0.2	
Insulin (pmol/l)	43.2 ± 6.5	30.3 ± 4.7	30.9 ± 3.2	40.1 ± 7.1	
Glucose OGTT (AUC)	843^*^	943	846^***^	991	^*^(Age)
Insulin OGTT (AUC)	37264	38931	34623	39010	
**CHOLESTEROL**					
Cholesterol (mmol/l)	4.4 ± 0.3^*^	5.4 ± 0.3	3.9 ± 0.1^**^	5.3 ± 0.3	^****^(Age)
HDL (mmol/l)	1.7 ± 0.1^§^	1.9 ± 0.2	1.3 ± 0.1^*^	1.7 ± 0.1	^***^(Gender)
					^*^(Age)
LDL (mmol/l)	2.3 ± 0.2^*^	3.2 ± 0.3	2.3 ± 0.1^**^	3.3 ± 0.3	^**^(Age)

*Subject characteristics in old and young females and males. Data are means ± SD*.

* *P < 0.05*,

** *P < 0.01*,

*** *P < 0.001*,

**** P < 0.0001 (Between age for each gender)

§ *P < 0.05*,

§§ *P < 0.01*,

§§§ *P < 0.001*,

§§§§ *P < 0.0001 (between gender for each age group). A separate column shows the overall effect with a two-way ANOVA (same significance levels as between age)*.

### *In vivo* study 2

Eight men with prostate cancer undergoing medical clinical castration, in form of the LHRH-agonist, Goserelin (Zoladex) were recruited from the Departments of Urology at Rigshospitalet and Herlev Hospital, as previously described (Hvid et al., 2013). Briefly, all subjects were treated with Goserelin for at least 3 months before inclusion in the study and continued the treatment throughout the study. Subjects were excluded if they had any infections 2 weeks before the study started or if they had severe health threatening metastases. Eight healthy men with normal testosterone levels, matched for age and VO_2_max, were recruited as a control group. Subject characteristics are listed in Table 2. On the experimental day subjects arrived in the morning in a fasted state. Testosterone levels were measured before an oral glucose tolerance test, 3–5 days pre and post-training.

**Table 2:** ***In vivo* study 2**.

	**Control**	**LHRH-agonist**
	**(*n* = 8)**	**(*n* = 8)**
Age	67.5 ± 2.9	67.9 ± 6.8
Weight (kg)	80.5 ± 12.7	80 ± 13.8
Height (cm)	177.6 ± 4.5	176.1 ± 9.4
**BODY COMPOSITION**		
BMI	25.5 ± 3.3	25.7 ± 3.6
Lean body mass pre-training (kg)	57.27 ± 2.1	52.15 ± 2.5
Lean body mass post-training (kg)	57.32 ± 2.07	52.13 ± 2.4
Fat mass pre-training (kg)	20.2 ± 2.9	24.5 ± 3.8
Fat mass post-training (kg)	19.3 ± 2.7	23.19 ± 2.9
**FITNESS**		
VO2 max pre-training (l/min/kg)	27 ± 3.8	25.3 ± 4.4
VO2 max post-training (l/min/kg)	31.3 ± 3.3^****^	29.4 ± 3.9^****^
**HORMONES**		
Testosterone (nmol/l)	13.5 ± 5.1^§§§^	0.5 ± 0.4

*Subject characteristics in LHRH-agonist treated prostate cancer patients and healthy controls. Fitness, lean body mass and fat mass significance levels were calculated with a Two-Way ANOVA RM and bonferroni's multiple comparison test was used as post-hoc test to identify any differences between groups before and after training*.

**** *P< 0.0001, symbolize overall effect of training. The remaining significance levels were calculated with unpaired t-tests*.

§§§ *P < 0.001 (between the two groups). Data are means ± SD*.

### Training protocol *in vivo* study 2

Subjects from *in vivo* study 2 performed and endurance training program for 12 weeks on a cycle ergometer (Monark 839E, Monark Ltd, Varberg, Sweden) consisting of three training sessions per week. All training sessions consisted of 5 min of warm up, 35 min of interval training and 5 min of cool down, as previously described (Hvid et al., 2013). The first 6 weeks the mean training intensity was at 65% of VO_2_max,and the last 6 weeks at 75% of VO_2_max. All training sessions were supervised and heart rate was continuously monitored (RS400; Polar, Kempele, Finland). The subjects were instructed to maintain their habitual diet and level of physical activity during the study. The subjects completed the diet and activity records for 3 days in the beginning of the training period and for 3 days at the end of the training period. Diet did not differ between groups.

### Body composition

Whole body fat and fat-free tissue mass measurements were performed using a dual-energy X-ray absorptiometry (DXA) scanner (Lunar Prodigy, GE Healthcare, WI, Madison USA, software v. 8.8) in both human *in vivo* studies.

### Muscle biopsies

Muscle biopsies from vastus lateralis were obtained at rest in the fasted state using the percutaneous needle method with suction under local anaesthesia, using 3–5 ml of 20 mg ml^−1^ lidocaine (SAD, Denmark Copenhagen). Muscle tissue was immediately frozen in liquid nitrogen and stored at −80°C until further analysis.

### Animal experimental protocols

Nine male 7 weeks old wild-type C57BL/6J mice originally obtained from Taconic, Denmark and inbred at the animal facility (Copenhagen, Denmark). All experiments were approved by The Animal Experiments Inspectorate of Denmark in accordance with the National Health and Medical Research Council of Australia Guidelines on Animal Experimentation. Mice were maintained on a 12-h light, 12-h dark cycle on standard rodent chow diet. Eight weeks old mice (*n* = 5) were surgically castrated, eliminating circulating testosterone. The control mice (*n* = 4) were sham operated to obtain equal treatment between groups. Twenty-four weeks post castration and sham operation the mice were sacrificed and quadriceps muscle was snap frozen in liquid nitrogen and stored at –80°C until further use. Quadriceps muscle was powdered in liquid nitrogen before RNA isolation (see separate procedure description).

### Cell experiments

Satellite cells were isolated from muscle biopsies as previously described (Scheele et al., 2012). Unless otherwise stated, all cell culture reagents were from Invitrogen, Carlsbad, CA, USA. Briefly, a muscle biopsy was obtained from vastus lateralis and digested in 5 ml HamF10 media containing 50% trypsin EDTA (1×) and 5 mg collagenase IV (Sigma-Aldrich, St. Louis, MO, USA) and BSA (Sigma-Aldrich) at 37°C for 5 min. The digestion solution was inactivated in serum on ice and the procedure was repeated. The digestion solution was filtered and centrifuged at 800 g for 7 min. Following washing with HamF10, cells were pre-plated in a 60 mm culture plate, to diminish fibroblast contamination. After 3 h incubation, the cell suspension was transferred to a 25 cm^3^ flask coated with matrigel (BD, Franklin Lakes, NJ, USA). The media, HamF10, 20% FBS, 1% Penicillin/Streptomycin and 1% Fungizone, was changed on day 4 after isolation and thereafter every second day. Cell cultures were expanded from the 25 cm^3^ flask to a 10 cm plate, which were assigned passage (P) 1. Thereafter, cells were split 1:3 at 75% confluence. Experiments were performed on cells at P6–P7. For differentiation, cells were seeded in DMEM low glucose (1 g/L), 10% FBS, 1% Penicillin/Streptomycin and, upon confluence, media was changed to DMEM high glucose (4.5 g/L), 2% horse serum, 1% Penicillin/Streptomycin. Experiments were performed on day 6 of differentiation by which time cells had fused into polynucleated myotubes. Differentiated myotubes were then incubated with 100 nM free testosterone 99% purum (Sigma-Aldrich, Switzerland) and 5α-androstan-17β-ol-3-one (Sigma-Aldrich, Switzerland) for 24 h on FBS free low glucose DMEM media to avoid contamination of testosterone present in the FBS. Testosterone and 5α-androstan-17β-ol-3-one were dissolved in Dimethyl Sulphoxide (DMSO) (Sigma-Aldrich, Switzerland) and thereafter mixed with DMEM media. The vehicle, DMSO, was added in equal concentrations as the testosterone stimulated cultures. All cell culture experiments were repeated 4 times (*n* = 4).

### RNA isolation

Approximately 30 mg skeletal muscle samples, powdered quadriceps muscle (mouse study) or *Vastus Lateralis* (human studies) were homogenized in TRIzol (Life Technologies) using a Tissuelyser (Qiagen, Valencia, CA, USA) and total RNA was isolated according to the manufacturer's protocol. Total RNA was dissolved in RNase-free water and quantified using a Nanodrop ND 1000 (Thermo Scientific, Wilmington, DE, USA).

### myomir quantification

The abundance of mature miRNAs was measured using TaqMan miRNA assays specific for miR-1 (Assay ID: 002222), miR-133a (Assay ID: 002246), miR-133b (Assay ID: 002247) and −206 (Assay ID: 000510) according to the manufacturer's directions (Applied Biosystems Inc., Foster City, CA, USA). Briefly, reverse transcription was performed with miRNA-specific RT primers and 10 ng of total RNA for 30 min at 37°C followed by 10 min incubation at 95°C. Quantitative real-time PCR (qPCR) was performed using the ViiA7 Sequence Detection System (Applied Biosystems) according to the manufacturer's protocol in triplicates, using the RT product and miRNA-specific PCR primers for 40 cycles. The final miRNA specific primer assay volume was 5% (20×) of the total volume for the qPCR, as recommended by the manufacturer's protocol (Applied Biosystems). RNU48 (Assay ID: 001006), a small nuclear RNA, and 18S was measured using the same type of detection assay (Applied Biosystems), and was used as an endogenous control for miRNA expression analyses in the human *in vivo* studies. Endogenous controls were tested for systematically variation between samples before further analysis. In the mouse study 18S (Mm03928990_g1) was used as an endogenous control, as RNU48 is not expressed in mice. RNU48 was used as endogenous control in human *in vivo* study 2 and in the human primary myotube *in vitro* study. In the human *in vivo* study 1, 18S expression was used as an endogenous control due to differentially expression of RNU48 between elderly and young subjects. Expression levels were calculated with the standard curve method. PCR efficiencies, linearity slopes, cycle threshold ranges and concentrations for the qPCR primers used in the study are listed in Table 3.

**Table 3:** **PCR standard curve data**.

	***In vivo* study 1 (Figure 1)**				***In vivo* study 2 (Figure 3)**			
**Primers**	**Lin. slope**	**Efficiency (%)**	**Range (CT)**	**Primer conc.**	**Lin. slope**	**Efficiency (%)**	**Range (CT)**	**Primer conc.**
miR-1	−3.12	106	18.3–22.3	20X	−3.55	94	21.6–24.9	20X
miR-133a	−3.18	104	18.6–22.7		−3.22	103	20.4–23.3	
miR-133b	−3.49	95	19.6–22.9		−3.27	102	19.7–22.7	
miR-206	3.5	95	21.6–24.6		−3.21	104	23.2–26.2	
RNU48	-	-	-	-	−3.38	98	27.4–30.5	
MYOD	-	-	-	-	−3.42	97	24.9–29.0	F: 300 nM R: 300 nM
MYOG	-	-	-	-	−3.15	105	22.7–26.5	
MYF-5	-	-	-	-	−3.28	101	25.5–29.3	
AR	-	-	-	-	−3.51	95	23.7–28.0	
18S	−3.26	102	14.7–18.5	F: 300 nM R: 300 nM	−3.44	97	13.0–17.2	
	**Mice study (Figure 4)**					***In vitro* study (Figure 5)**				
miR-1	−3.28	101	18.1–21.8	20X	−3.47	96	28.1–32.3	20X
miR-133a	−3.35	99	16.6–20.7		−3.59	92	24.9–29.2	
miR-133b	−3.35	99	17.2–21.3		3.46	96	24.5–28.7	
miR-206	−3.41	97	24.4–27.5		−3.49	95	22.9–27.1	
RNU48	-	-	-	-	−3.16	105	26.8–30.7	
MYOD/Myod	−3.25	102	22.8–26.8	F: 300 nM R: 300 nM	−3.47	96	22.7–26.9	F: 300 nM R: 300 nM
MYOG/Myog	−3.83	88	25.3–29.1		−3.36	99	20.5–24.5	
MYF-5/Myf-5	−3.8	84	26.1–31.0		−3.53	94	22.5–26.8	
AR/Ar	−3.19	104	22.9–25.8		−3.46	96	27.1–31.2	
18S	−3.36	99	13.4–17.4		−3.5	95	13.7–18.1	

PCR efficiency, linearity slope, cycle threshold range and concentration for each primer used in the study.

### mRNA quantification

Total RNA (0.5 μg) was reverse-transcribed using cDNA high capacity kit (Applied Biosystems) according to the manufacturer's protocol. cDNA samples were loaded in triplicate and qPCR was performed using ViiA7 Sequence Detection system (Applied Biosystems, Foster City, CA, USA) according to the manufacturer's protocol using either SYBR chemistry or primer-probe based pre-optimized detection methods. Primers were designed using Roche Applied Science Assay Design Center (Roche).

#### Human primers

*AR* (CT-range: 23.1–24.5)

Pre-optimized assay from Applied Biosystems (Hs00171172_m)

*MYOD* (CT-range: 24.2–26.2)

Forward primer—CACTACAGCGGCGACTCC

Reverse primer—TAGGCGCCTTCGTAGCAG

*MYOG* (CT-range: 21.9–23.9)

Forward primer—GCTCAGCTCCCTCAACCA

Reverse primer—GCTGTGAGAGCTGCATTCG

*MYF5* (CT-range: 24.7–26.8)

Reverse primer—CTATAGCCTGCCGGGACA

Reverse primer—TGGACCAGACAGGACTGTTACAT

#### Mouse primers

*Ar* (CT-range: 21.3—23.2)

Forward primer—TCGGTGGAAGCTACAGACAA

Reverse primer—TAGACCCTTCCCAGCCCTAA

*Myog* (CT-range: 24.1–27.2)

Forward primer—GGTGCCCAGTGAATGCAACT

Reverse primer—AGCCGCGAGCAAATGATCT

*Myod* (CT-range: 22.6–25.2)

Forward primer—CACTCCGGGACATAGACTTGACA

Reverse—CCCCGACGGCTCTCTCTG

*Myf5* (CT-range: 26.3—29.1)

Forward primer—GCCAGTTCTCCCCTTCTGAGTS

Reverse primer—ACTGGTCCCCAAACTCATCCT

Pre-optimized assays from Applied Biosystems for 18S rRNA (Human, Hs99999901_s1. Mouse, Mm03928990_g1) was used as endogenous control. To assess amplification efficiency, two fold serial dilutions were performed for all primers. Based on these, cDNA was diluted 1:10 for Myog, MyoD, Myf-5, and Ar and 1:200 for 18S, to ensure linear amplification. Melting curve analysis was performed to ensure primer specificity for SYBR green reactions. Expression levels were calculated with the standard curve method (Table 3).

#### Statistical analysis

**Figure 1:**
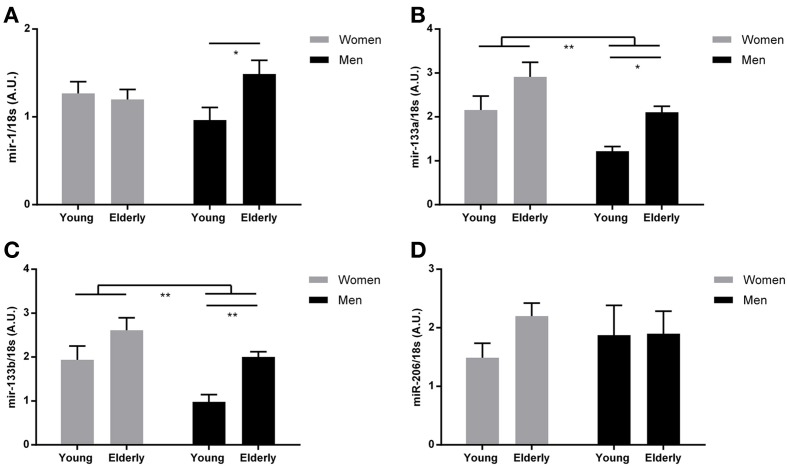
**MyomiR expression in human skeletal muscle from elderly and younger individuals**. Muscle biopsies were obtained from a cohort of healthy elderly and younger men and women (*n* = 9 in each group) and myomiR levels were measured by using qPCR. An ordinary Two-Way ANOVA revealed an overall effect of both gender and age on miR-133a and miR-133b expression **(B,C)** (^**^P < 0.01) and a significant interaction for miR-1 expression (^**^P < 0.01). When using Bonferroni multiple comparison *post-hoc* test it was demonstrated that miR-1 **(A)**, miR-133a **(B)**, and miR-133b **(C)** expression levels were higher in elderly compared to younger men (^*^P = 0.02, ^*^P = 0.03, ^***^P = 0.008, respectively) There was no effect of age or gender on mir-206 expression **(D)** (*P* > 0.05). Data are mean ± SEM.

**Figure 2:**
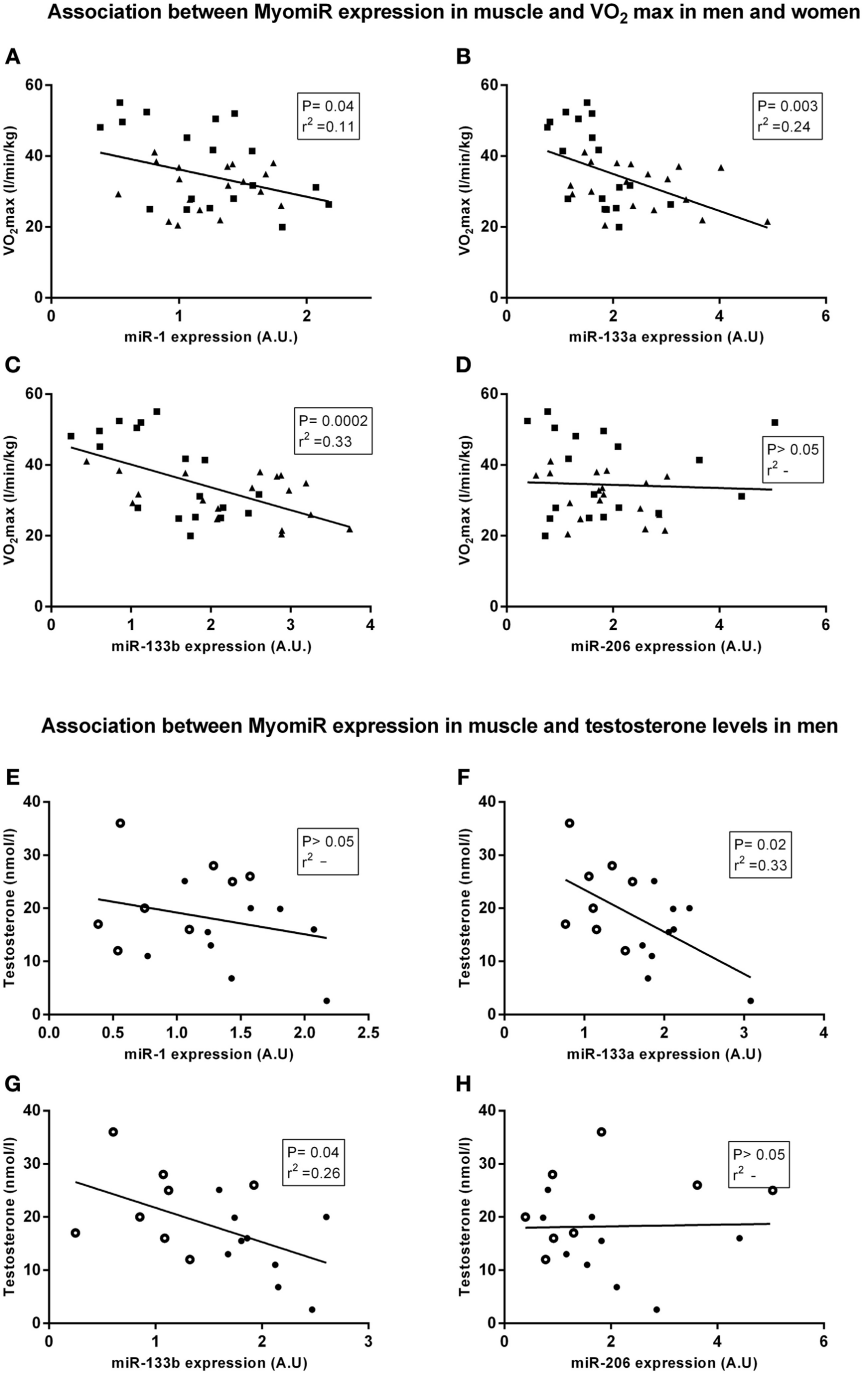
**Linear regression analysis between myomiR expression and physiological characteristics**. The cohort described in Figure 1 was assessed for associations between muscular miRNA expression and physiological characteristics. In panel **(A–D)** are men symbolized (■) and women are symbolized (▼). In panel **(D,E)** observations from elderly men are symbolized (•) and observations from younger men are symbolized (○). miR-1, miR-133a, and miR-133b **(A–C)** were inversely correlated with maximal oxygen uptake in women and men (*n* = 36) (*P* < 0.05, 0.01 and 0.001, *R*^2^ = 0.11, 0.24, and 0.33). In addition, miR-133a **(F)** and miR-133b **(G)** expression were negatively correlated with testosterone levels in men (*n* = 18) (*P* < 0.05, *R*^2^ = 0.33 and *P* < 0.05, *R*^2^ = 0.26). miR-1 **(E)** and miR-206 **(H)** expression were not correlated with testosterone in men. Data are mean ± s.e.m.

**Figure 3:**
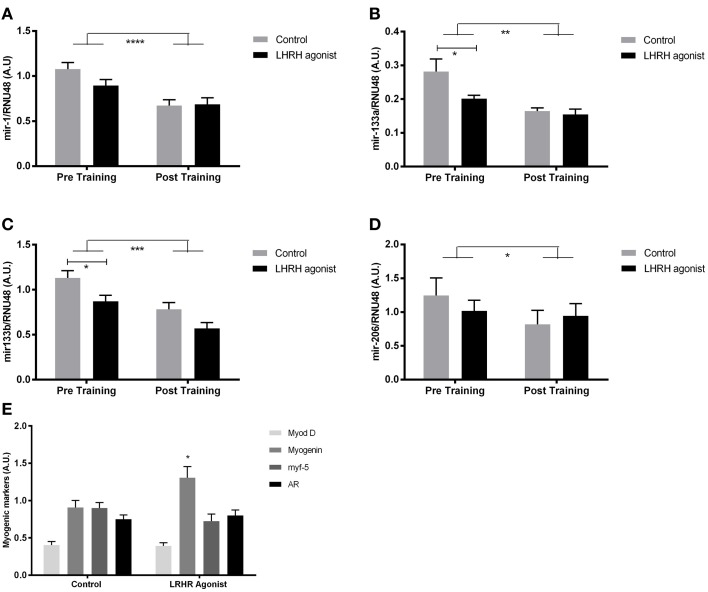
**MyomiR expression in testosterone depleted men before and after training**. MyomiR expression in the skeletal muscle of LHRH agonist treated prostate cancer patients (*n* = 8) and healthy controls (*n* = 8), in resting samples prior to and following 12 weeks of aerobic exercise training. A Two-Way ANOVA (RM) (miR-1, ^****^P < 0.0001. miR-133a, ^**^P < 0.01. miR-133b, ^***^P < 0.001, miR-206 ^*^P < 0.05). Bonferroni multiple comparison *post-hoc* tests revealed a significant lower expression of mir-133a **(B)** (^*^P = 0.02) and mir-133b **(C)** (^*^P = 0.03), but not mir-1 **(A)** or miR-206 **(D)** in testosterone blocked patients at rest before training. The expression of myomiRs in testosterone blocked patients and healthy controls were identical after the training period. The expression of MYOG **(E)** (^*^P < 0.05) at rest was upregulated in testosterone-blocked participants. No regulation of MYOD, MYF-5, or AR was detected. Data are mean ± SEM.

**Figure 4:**
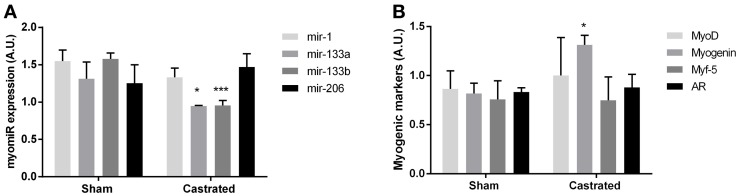
**Muscle of castrated (*n* = 5) and sham operated (*n* = 4) C57BL/6J mice**. An unpaired t-test demonstrated a significant lower expression of mir-133a (^*^P < 0.05) and mir-133b (^***^P < 0.001) in the skeletal muscle of castrated mice. The expression of mir-1 and mir-206 were not significantly different between castrated and sham operated mice **(A)**. Whereas the expression of Myog was upregulated (^*^P< 0.05) in castrated mice, no regulation of Myod, Myf-5, or Ar was detected (*P* > 0.05) **(B)**. Data are mean ± SEM.

**Figure 5:**
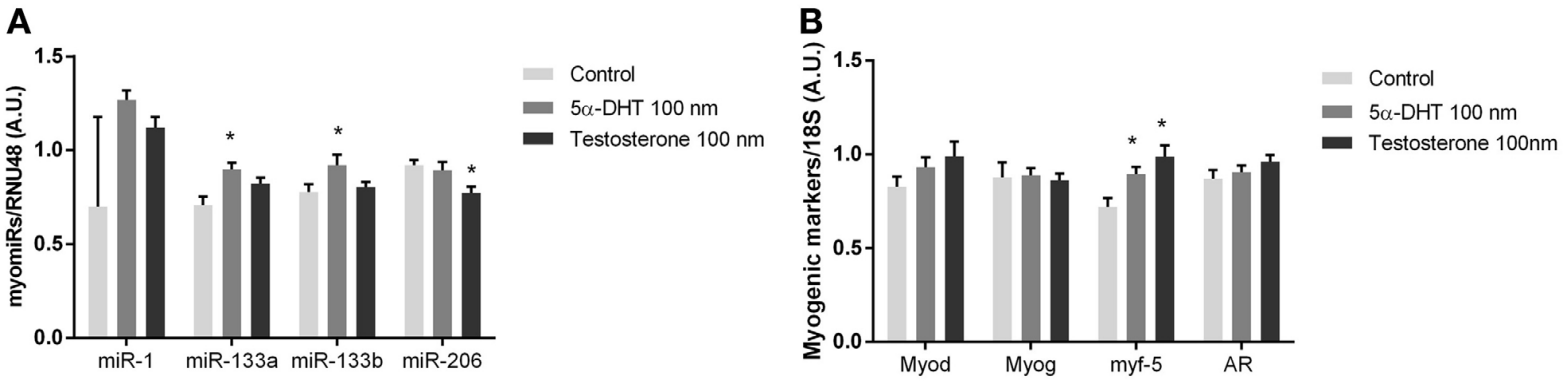
**myomiR expression in testosterone treated human muscle cells**. myomiR expression in differentiated human primary myocytes in response to 100 nM testosterone (*n* = 4), and 5α-DHT (*n* = 4). Control cultures (*n* = 4) were added DMSO in same concentrations as testosterone and 5α-DHT stimulated myocytes. miR-133a/b was upregulated in response to 5α-DHT treatment and mirR-206 expression was downregulated in response to to testosterone treatment (^*^P < 0.05) **(A)**. Whereas the expression of Myf-5 was upregulated in response to 5α-DHT and testosterone (^*^P < 0.05) no regulation of Myod, Myog or AR was detected **(B)**. Data are mean ± SEM.

The statistical analysis was performed using GraphPad Prism 6.0 (GraphPad Software Inc., La Jolla, CA, USA). Results in the figures are presented as means ± SEM. Data in the tables are presented as means ± SD. All data were tested for normality of distribution before further analysis using the Kolmogorov–Smirnov test. An ordinary Two-Way ANOVA model 1 was used to analyse the data in Figure 1 and Table 1 (age and gender as main effects) and Two-Way ANOVA repeated measures was used for Figure 3. Bonferroni's multiple comparisons test was used as *post-hoc* tests to identify differences expression within the two gender groups when the Two-Way ANOVA tests reached significant level *P* < 0.05. Unpaired *t*-tests were used to analyse the data in the mice study (Figure 4), the cell culture study (Figure 5) and the pre-training expression levels of the myogenic markers (MYOD, MYOG, MYF5) and the androgen receptor (AR) (Figure 3). Linear regression analysis was applied as stated in the legends Figure 2, *P* < 0.05 was used as significance level.

## Results

### Gender and age affects miR-133a and miR-133b expression

To examine whether age and/or gender affects myomiR expression in skeletal muscle, we measured myomiRs at rest in biopsies taken from vastus lateralis in young and elderly men and women (Figure 1). An ordinary Two-Way ANOVA revealed a markedly effect of both gender and age on miR-133a and miR-133b expression (*P* < 0.01), where both factors are associated with an overall higher expression of both mature miRNA transcripts. Where no effect of age and/or gender on miR-1 and miR-206 expression was detected (*P* > 0.05), a significant interaction on miR-1 expression was identified with a Two-Way ANOVA test (*P* < 0.05). As expected, muscle mass was clearly lower in the women (Table 1). Interestingly, an association between increased muscle mass and downregulation of myomiRs has been reported (McCarthy and Esser, 2007). Other variables that might be related to myomiR expression included aerobic fitness and testosterone levels, which differed between the four groups (Table 1) and were therefore selected for additional examination.

### miR-1, miR-133a and miR-133b expression is upregulated with aging in men

To address a potential involvement of miR-1, miR-133a, and miR-133b in the age-related decline in muscle function for both genders, we used a bonferroni multiple comparison *post-hoc* test.

We found an increased expression of miR-1 (*P* = 0.02), miR-133a (*P* = 0.03) and miR-133b (*P* = 0.008) in elderly men compared to younger men (Figures 1A–C). A Two-Way ANOVA revealed no effect of age, gender or neither any interaction (*P* > 0.05) on miR-206 expression and therefore no *post-hoc* test was performed within the gender groups (Figure 1D). No significant differences in expression of any of the three myomiRs in women were detected (*P* > 0.05) (Figures 1A–C). Interestingly, similar to the observed myomiR expression pattern, we identified age related differences in fasting and OGTT plasma glucose levels in men but not in women (Table 1).

### myomiR expression is negatively correlated with testosterone and fitness levels

In line with our previous findings (Nielsen et al., 2010) aerobic fitness in all subjects was negatively correlated with miR-1 and miR-133a and miR-133b (Figures 2A–C) (*P* < 0.05, 0.01 and 0.001, *r*^2^ = 0.11, 0.24, and 0.33, respectively). Furthermore, circulating testosterone levels in men was negatively correlated with miR-133a and miR 133b expression (Figures 2F,G) (*P* < 0.05, *r*^2^ = 0.33 and *P* < 0.05, *r*^2^ = 0.26). miR-206 expression in men was not correlated with testosterone levels or the subjects' maximal oxygen capacity. myomiR expression was not correlated with testosterone levels in women (data not shown). Thus, we conducted subsequent experiments addressing the potential role that circulating testosterone and/or aerobic fitness might play in regulating skeletal muscle miR-133a/b in men.

### miR-133a and miR-133b are down-regulated in testosterone blocked participants

To test whether myomiR expression was regulated by testosterone, aerobic exercise or both, we measured myomiR expression in vastus lateralis of LHRH-agonist treated prostate cancer patients and healthy controls matched for maximal oxygen consumption (Table 2). In addition, myomiR expression was measured after a 12 week aerobic exercise training programme to test if increased fitness level altered myomiR expression independent of plasma testosterone levels. Muscle biopsies were obtained at rest before and after the training period. Partly in line with our previous results (Nielsen et al., 2010), a Two-Way ANOVA (RM) demonstrated a main effect of training in terms of decreased expression in all four myomiRs (miR-1, *P* < 0.0001. miR-133a, *P* < 0.01. miR-133b, *P* < 0.0001, miR-206 *P* < 0.05). Surprisingly, a bonferroni multiple comparison test revealed reduced miR-133a/b expression (miR-133a, *P* = 0.02. miR-133b, *P* = 0.03.) before training in the LHRH-agonist treated participants with almost no plasma testosterone (Figures 3B,C). That observation was in opposition to the association observed in the elderly vs. young participants in study 1 (Figures 2F,G). miR-1 and miR-206 expression were not altered by LHRH-agonist treatment before the training period (*P* > 0.05) (Figures 3A,D). Regardless of pre-exercise intervention level, training equalized the expression of miR-133a and miR-133b between healthy and LHR-treated participants to a lower level. An overall effect of LHRH-agonist treatment was observed for miR-133a and miR-133b expression (*P* < 0.05), but not in miR-1 or miR-206 expression (*P* > 0.05). No significant interaction was observed in any of the four myomiRs, (*P* > 0.05). As mentioned in the introduction, testosterone may indirectly regulate myomiRs through increasing transcription of the MRFs MYF5, MYOD, and MYOG and the androgen receptor (AR) (Chen et al., 2006; Sweetman et al., 2008; Wannenes et al., 2008; Dubois et al., 2012; Nasipak and Kelley, 2012). We therefore measured mRNA levels of these genes at rest. Intriguingly, while most of the transcription factors remained unchanged the expression of MYOG was significantly higher in the LHRH-treated patients (Figure 3E).

### miR-133a and miR-133b are down-regulated in castrated mice

As our human model included subjects with cancer and were lacking appropriate controls, we were concerned about non-specific effects. Therefore, we also measured myomiR expression in skeletal muscle of castrated mice, which eliminated circulating testosterone, and sham operated control mice. Consistent with our findings in the LHRH-agonist treated men with low circulating testosterone, castrated mice had a lower expression of mir-133a (*P* < 0.05) and mir-133b (*P* < 0.001) (Figure 4A). The expression of mir-1 and mir-206 were identical in skeletal muscle in both castrated and sham operated mice (*P* > 0.05). Furthermore, the MRFs measured in castrated mice were unchanged with the exception of increased Myog mRNA levels, in line with the finding in our testosterone blocked human participants (*P* < 0.05) (Figure 4B).

### 5α-dihydrotestosterone regulates miR-133a, miR-133b, and miR-206 expression in human primary myocytes

To evaluate if testosterone directly regulates myomiR expression in skeletal muscle we established a cell culture model using human myotubes. Human myotubes were incubated with 100 nM of testosterone or 100 nM 5α-dihydrotestosterone (5α-DHT), which has a higher AR affinity than testosterone (Saartok et al., 1984). 5α-DHT is formed *in vivo* by 5α-reductase acting on testosterone intracellularly in myocytes (Sato et al., 2008). Whether testosterone is converted to 5α-DHT *in vitro* is unknown, and thus we incubated primary myotubes with either testosterone or 5α-DHT for 24 h (Sato et al., 2008). Consistent with the *in vivo* experiments suggesting a role for testosterone in regulating miR-133a/b, 5α-DHT incubation in culture increased miR-133a and miR-133b (*P* < 0.05) (Figure 5A). Surprisingly, incubation with 5α-DHT decreased miR-206 (*P* < 0.05), While MYF-5 was upregulated in response to testosterone or 5α-DHT (*P* < 0.05), AR, MYOG, and MYOD mRNA was not significantly altered (Figure 5B).

## Discussion

In the current study, we found that the expression of miR-1, miR-133a, and miR-133b was higher in the skeletal muscle of elderly compared to younger men and that miR-133a/b was higher expressed in women compared to men. miR-133a/b was inversely correlated with an age-dependent decrease of testosterone in men. However, our subsequent studies demonstrated that testosterone *positively* regulated miR-133a/b. Finally, our data suggest that increased physical activity compensates for the loss of testosterone in terms of myomiR regulation.

Our collective data from three independent models, indicate that testosterone up-regulates miR-133a, and 133b. Importantly, we also show that physical activity overrides the regulatory effect of testosterone on miR-133a/b expression. Our findings suggest that an age-dependent decline in testosterone and increase of miR-133a/b expression are independent events. It has been established that increased physical fitness is associated with a down-regulation of myomiRs (Nielsen et al., 2010; Keller et al., 2011). Therefore, it is likely that the decline in physical activity is the main determining factor involved in the age-dependent up-regulation of miR-1 and miR-133a/b.

The observed effects of testosterone and age as inducers of miR-133a and miR-133b expression are seemingly in opposition. However, in addition to miR-133a/b, miR-1 was induced in the elderly group. miR-1 has a specific set off predicted mRNA targets and the regulatory net-effect will thus differ from the effect of testosterone-mediated miRNA regulation. It is therefore possible that myomiR alterations contribute to age-dependent decline in muscle function. Interestingly, our human data suggests that the effects of testosterone on myomiR regulation are minor compared to the effect of increased physical activity. Androgenic control of miR-133a/b expression therefore seems to be a separate regulatory mechanism that may play a role during certain physiological conditions, such as physical inactivity.

Studies have shown that alterations in myomiR expression are associated with both hypertrophy and atrophy in skeletal muscle (Care et al., 2007; McCarthy and Esser, 2007). Importantly, age related loss of muscle mass has been extensively described in the literature, including the age intervals for the subjects in the current study (Forbes and Reina, 1970). However, our elderly subjects were healthy and we did not observe any age related differences in lean body mass within each gender group in the current study. Therefore, changes in myomiR expression might represent an early event preceding potential loss in muscle mass. Noteworthy, lean body mass is assumed to reflect the muscle mass of the subjects but this assumption is subject to some degree of uncertainty. A study by Loon and co-workers revealed that muscle cross sectional area and muscle fiber type size were markedly decreased in older subjects compared to young. However, differences in lean body mass between young and old when performing DXA scan measurements, was not detectable (Nilwik et al., 2013). Determination of lean body mass by DXA is an indirect quantification method and not a direct measurement of the actual muscle mass or cross sectional area, two physiological parameters directly related to muscle function. Therefore, it cannot be excluded that our older participants in fact have suffered from a loss of muscle function that we were not able to detect in the present cross-sectional study. In contrast to the age dependent up-regulation of myomiRs in men we did not observe a similar regulation pattern in women (Figure 1). A possible explanation for this could be less distinct differences in aerobic fitness between the elderly and younger women. Interestingly, the women had a markedly increase in miR-133a and miR-133b expression compared to men. Emphasizing the gender differences of skeletal muscle a recent study by Liu and co-workers demonstrated that the global transcriptome in muscle of older men and women differed markedly for groups of genes important for muscle function (Liu et al., 2013).

A few previous studies have investigated the age dependent miRNA alteration in human skeletal muscle. Global miRNA expression in the skeletal muscle of young vs. elderly men was compared using a miRNA microarray by Drummond et al. (2011). Drummond found that 18 miRNAs, including miR-133a and miR-133b, were differentially expressed. In contrast to our results from the current study, their microarray data pointed toward a downregulation of miR-133a/b in elderly men. Interestingly, Drummond et al. have also reported that the precursors to mature miRNAs, pri-miR-1, and pri-miR-133a, but not the mature forms, were increased in skeletal muscle of elderly men compared to younger controls (Drummond et al., 2008b). A number of reasons could explain the discrepancy with our data. In contrast to the study by Drummond et al. the testosterone levels in the current study differed between young and elderly men (Drummond et al., 2008a). Furthermore, aerobic fitness levels were not determined and our young subject group is approximately 8 years younger than in the study performed by Drummond and co-workers (Drummond et al., 2008a; Nielsen et al., 2010).

It is well known that miRNAs frequently are co-expressed in clusters with neighboring miRNAs (Baskerville and Bartel, 2005). However, in the current study we observe a co-regulation of myomiRs from separate loci in response to alterations in circulating testosterone levels. Interestingly, steroid hormonal post-transcriptional control of miRNA expression in several cell types, including muscle, has been described in the literature (Yamagata et al., 2009). Thus, it is possible that the regulation of mir-133a/b expression by testosterone occurs through a post-transcriptional processing of the pri- and/or pre-miRNA transcripts.

Our human participants depleted for testosterone were recently diagnosed with prostate cancer and on LHRH agonist therapy. Some limitations with this model exist and should be discussed. For instance, each subject was not his own control since ethics dictated that treatment was started immediately after the diagnosis excluding that we could apply a longitudinal study design. Furthermore, differences between the control and treated group could be due to the cancer rather than the lack of circulating testosterone. Therefore, we included two additional models to verify our findings from the human *in vivo* study; castration of male mice which also eliminates circulating testosterone and stimulation of human myotubes *in vitro*. These two models both confirmed our initial findings in the human *in vivo* model. We thus show a physiological role of testosterone in the regulation of miR-133a and miR-133b in human and murine skeletal muscle.

In this study we have examined the myomiR expression in resting skeletal muscle of elderly and younger men and women along with the role of testosterone and physical fitness on muscle myomiR regulation. We clearly show that alterations in testosterone levels are sufficient to alter myomiR expression. We furthermore conclude that aerobic exercise training is likely to independently regulate myomiR and seem to override any effects of testosterone.

## Author contributions

Study conception and design: Søren Nielsen, Matthew J. Laye, Bente K. Pedersen. Collection, analysis and interpretation of data: Søren Nielsen, Matthew J. Laye, Bente K. Pedersen, Thine Hvid, Camilla Scheele, Meghan Kelly, Christine Dethlefsen, Neha Mathur, Kamilla Winding, Birgitte Lindegaard. Drafting the article or revising it critically for important intellectual content:: Søren Nielsen, Matthew J. Laye, Bente K. Pedersen, Thine Hvid, Camilla Scheele, Meghan Kelly, Christine Dethlefsen, Neha Mathur, Kamilla Winding, Birgitte Lindegaard. All authors approved the final version for publication.

### Conflict of interest statement

The authors declare that the research was conducted in the absence of any commercial or financial relationships that could be construed as a potential conflict of interest.
